# Nitroprusside Combined with Leg Raise at the Time of Right Heart Catheterization to Differentiate Precapillary from Other Hemodynamic Forms of Pulmonary Hypertension: A Single-Center Pilot Study

**DOI:** 10.3390/jcdd11040124

**Published:** 2024-04-19

**Authors:** Mostafa Naguib, Ahmed Aljwaid, Dean Marella, Raul J. Flores, Abhishek Singh

**Affiliations:** 1 Department of Internal Medicine, Morristown Medical Center, Morristown, NJ 07960, USA; 2 Department of Cardiology, Gagnon Cardiovascular Institute, Morristown Medical Center, Morristown, NJ 07960, USA; 3 Heart Success Program (Advanced Heart Failure Program), Department of Cardiology, Gagnon Cardiovascular Institute, Morristown Medical Center, Morristown, NJ 07960, USA

**Keywords:** combined pre- and postcapillary pulmonary hypertension, nitroprusside, leg raise

## Abstract

Pulmonary hypertension (PH) can arise from several distinct disease processes, with a percentage presenting with combined pre- and postcapillary pulmonary hypertension (cpcPH). Patients with cpcPH are unsuitable candidates for PH-directed therapies due to elevated pulmonary capillary wedge pressures (PCWPs); however, the PCWP is dynamic and is affected by both preload and afterload. Many patients that are diagnosed with cpcPH are hypertensive at the time of right heart catheterization which has the potential to increase the PCWP and, therefore, mimic a more postcapillary-predominant phenotype. In this small pilot study, we examine the effect of nitroprusside combined with dynamic preload augmentation with a passive leg raise maneuver in hypertensive cpcPH patients at the time of right heart catheterization to identify a more precapillary-dominant PH phenotype. Patients that met the criteria of PCWP ≤ 15 mmHg with nitroprusside infusion and PCWP ≤ 18 mmHg with nitroprusside infusion and simultaneous leg raise were started on pulmonary vascular-targeted therapy. Long-term PH therapy was well tolerated, with increased six-minute walk distance, improved WHO functional class, decreased NT-proBNP, and improved REVEAL 2.0 Lite Risk Score in this precapillary-dominant PH phenotype. This small study highlights the importance of characterizing patient physiology beyond resting conditions at the time of right heart catheterization.

## 1. Introduction

Pulmonary hypertension (PH) is defined by an abnormal elevation in the mean pulmonary artery pressures (mPAP) which can result from several distinct disease processes. The most common type of PH is World Health Organization (WHO) Group II or PH caused by predominant left heart disease [1,2]. In contrast, WHO Group 1 PH, or PH due to pulmonary vascular disease also known as pulmonary arterial hypertension (PAH), is rare [1,3]. Patients with PAH face a high degree of morbidity and mortality, with end-stage illness characterized by RV-PA uncoupling and resultant RV failure. Treatment for PAH is focused on interventions aimed at decreasing PVR to alleviate this RV-dependent circulatory limitation.

A critical step in diagnosis is invasive hemodynamic assessment utilizing right heart catheterization (RHC) as the gold standard method to diagnose PH. RHC allows for hemodynamic “phenotyping” of the different PH subgroups. In the setting of an elevated pulmonary vascular resistance (PVR) > 3 WU, the pulmonary capillary wedge pressure (PCWP) becomes the “gatekeeper” that differentiates pre- (PCWP ≤ 15 mmHg) from postcapillary (PCWP > 15 mmHg) PH [1]. Approximately 14% of patients with PH due to left heart disease develop combined pre- and postcapillary PH (cpcPH) with PVR ≥ 3 and PCWP > 15 mmHg [4]. Patients with this hemodynamic phenotype share many similarities to patients with PAH, including higher mortality [2,5,6]. In addition, patients with precapillary disease can develop a postcapillary component over time, i.e., due to the development of HFpEF, valvular disease, or infiltrative disease, which may present as a cpcPH phenotype. Pulmonary vascular-targeted treatment is limited in cpcPH due to an elevated PCWP resulting in the inability to unload the left heart at rest or with exertion [6]. 

Accurate assessment of the PCWP during RHC is therefore crucial to differentiate which patients could benefit from PAH-targeted therapy. In making these assessments, it is important to remember the PCWP is a dynamic surrogate value that can be affected by altered preload and afterload. Dynamic testing utilizing vasoactive medications or maneuvers such as exercise, fluid administration, or passive leg raise have become increasingly utilized in the phenotypic assessment of patients with PH [7,8,9,10,11,12,13,14]. By examining filling pressures in conditions other than rest, one can potentially reach a more nuanced assessment of patient physiology and identify candidates for PH therapies that would otherwise go unrecognized.

Systemic hypertension present at the time of RHC, including in those that do not have a diagnosis of hypertension or are normally well controlled, may cause a temporary rise in the PCWP that will mimic a hemodynamic phenotype that meets criteria for cpcPH. However, the PCWP may normalize with improved control of the LV afterload, unmasking a precapillary pulmonary hypertension phenotype. This group of patients whose PCWP normalizes represents a group that may benefit from pulmonary vascular therapy. Nitroprusside, a rapidly acting balanced vasodilator, is an attractive agent in this clinical situation. Nitroprusside can quickly decrease LV afterload to assess the ability to reduce PCWP. Studies examining the use of nitroprusside in patients with PH have been primarily limited to risk stratification for heart transplantation to assess PVR reversibility and to optimize LV–arterial coupling [15,16,17]. It has also been used in several studies in HFpEF and PAH [18,19]. The potential flaw of nitroprusside is the simultaneous venodilator effect that can reduce preload and contribute to PCWP reduction. This venodilating effect can be mitigated by simultaneous preload augmentation with passive leg raise. Passive leg raise has been utilized as a form of dynamic preload augmentation in HFpEF and PH [20,21]. Passive filling via gravity from venous reservoirs in the legs can replicate a state of increased venous return of ~300 mL of blood in the recumbent patient as a means of assessing vascular and ventricular compliance [22]. In fact, a PCWP ≥ 19 mmHg with passive leg raise has been shown to have 100% specificity for diagnosing occult HFpEF, independent of the use of diuretics [21]. Passive leg raise has the advantage of transiently increasing the cardiac preload without actively administering fluids.

**Figure 1:**
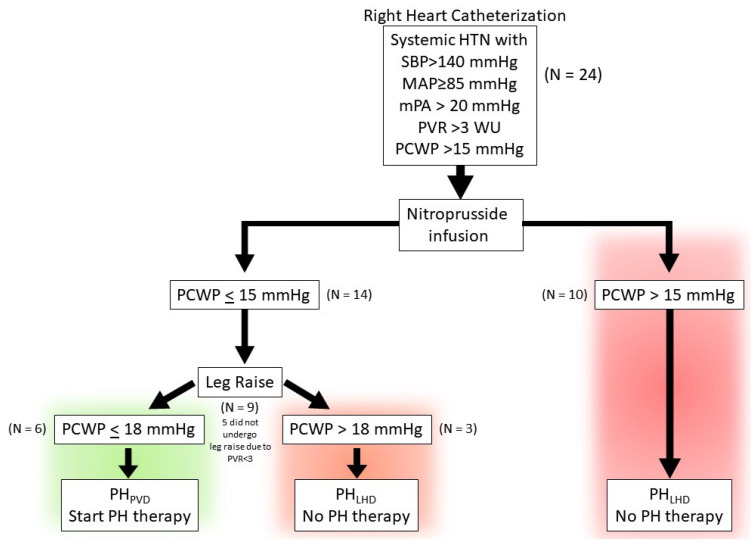
Schematic depiction of the study protocol and phenotyping of patients.

In this small pilot study, we evaluate the effect of combining nitroprusside infusion with passive leg raise during RHC to identify patients with predominant precapillary pulmonary hypertension which may be masked by a temporary rise in the PCWP due to systemic hypertension (Figure 1). The inability to decrease PCWP to ≤15 mmHg with nitroprusside infusion is suggestive of predominant postcapillary PH (defined here as PH_LHD_) in a patient presenting as cpcPH, and identifies unsuitable candidates for pulmonary vascular-targeted therapy. In contrast, a decrease in the PCWP to ≤15 with nitroprusside infusion may identify predominant precapillary PH (defined here as PH_PVD_). Addition of dynamic preload augmentation via passive leg raise at the time of nitroprusside infusion in this PH_PVD_ group can mitigate the venodilating effect of nitroprusside and help identify patients that may tolerate pulmonary vascular therapy. We hypothesize that a patient presenting with systemic hypertension with cpcPH to the cardiac catheterization lab may tolerate PH therapy if the following occur:

(1) the PCWP decreases to ≤15 mmHg and PVR remains ≥3 with nitroprusside infusion, suggestive of predominant precapillary PH.
(2) the PCWP remains ≤18 mmHg with nitroprusside infusion and passive leg raise.

Patients that met the above criteria were initiated on pulmonary vasodilator therapy and followed.

## 2. Study Design

The study is a retrospective review of 24 consecutive patients referred for pulmonary hypertension evaluation who underwent RHC with nitroprusside challenge by the PH program at a large community hospital network between September 2022 and December 2023. All patients included in the study were noted to have systemic hypertension during the RHC, even if they did not have a diagnosis of systemic hypertension, and met criteria for pulmonary hypertension (PVR ≥ 3 WU and mean pulmonary artery pressure [mPAP] ≥ 20 mmHg). Nitroprusside is routinely administered in our laboratory when PCWP is elevated to assess reversibility. Patients with LVEF > 50% were included if they were hypertensive during the RHC on the cardiac catheterization table with a systolic blood pressure >140 mmHg and mean arterial pressure (MAP) ≥ 85 mmHg. Patients were excluded if they had LVEF < 50%, significant left-sided valvular disease (i.e., >moderate mitral regurgitation/aortic stenosis/aortic regurgitation), congenital heart disease, cardiac transplantation, and infiltrative, restrictive, or hypertrophic cardiomyopathies. The patients were grouped based on the PCWP response to nitroprusside (Figure 1). If the PCWP decreased to ≤15 mmHg with nitroprusside, the patient was assigned to the PH_PVD_ group, and if it remained >15 mmHg despite nitroprusside, then the patient was assigned to the PH_LHD_ group (Table 1). The study protocol was reviewed by the Atlantic Health System institutional review board and found to be exempt from the regulations that govern human subject research (IRB submission # 2139071-1).

### 2.1. Right Heart Catheterization and Nitroprusside Protocol

Patients were studied receiving chronic medications in an optimized non-decompensated fasted state in the supine position without sedation. Standard right heart catheterization was performed through the internal jugular vein. All measurements were performed at end-expiration over several cardiac and respiratory cycles. End-expiratory mean PCWP and RAP were determined via mean mid-A wave if the patient was in normal sinus rhythm (NSR) and mid-C (when visible) or pre-V for patients in atrial fibrillation. All measurements were adjudicated by one investigator (A.S.) from electronically stored recordings of pressure tracings. Systemic arterial blood pressure (BP) was measured by cuff sphygmomanometry. Cardiac output was determined by the estimated Fick or by thermodilution method. After baseline hemodynamic data were acquired, sodium nitroprusside was administered at incremental doses starting at 0.25 to 0.5 μg/kg/min, titrated to the following: (1) PCWP ≤ 15; (2) MAP ≤ 85 mmHg; or (3) patient intolerance (e.g., lightheadedness). Hemodynamic measurements were then repeated (Figure 1). For those patients whose PCWP ≤ 15 mmHg and PVR ≥ 3 WU, a leg raise was performed to assess if the PCWP increased above 18 mmHg as a surrogate for left heart noncompliance that could potentially limit PAH therapy [21]. In total, 5 of 14 patients with PCWP ≤ 15 mmHg post nitroprusside did not undergo leg raise as the PVR < 3 WU. Hemodynamics were repeated <2 min post leg raise as the effect can rapidly dissipate [22]. 

### 2.2. Statistical Analysis

Continuous variables are reported as mean ± standard deviation. The categorical variables are reported as absolute numbers and proportions where applicable. For baseline characteristics, differences among groups were verified by Wilcoxon matched pairs or Mann–Whitney comparison for continuous variables and by chi-square test (or Fischer test) for categorical variables. An ANOVA and Kruskal–Wallis test were used to compare the differences among groups for normally and non-normally distributed variables, respectively, using Prism version 10.1.2 (GraphPad Software, Boston, MA, USA, www.graphpad.com (accessed on 20 December 2023)).

## 3. Results

### 3.1. Baseline Demographic Data and Clinical Characteristics Are Summarized in Table 1

The patients represent a typical elderly, overweight, and predominantly female combined pre- and postcapillary pulmonary hypertension population. There was no significant difference in comorbidities or medications between the groups, with the exception of sodium–glucose cotransporter-2 inhibitor (SGLT2i). The PH_LHD_ were on SGLT2i at a higher rate than PH_PVD_ (*p* = 0.0104). 

Although there was no significant difference between the two groups with respect to echo parameters indicative of HFpEF, such as enlarged LAVi or the presence of diastolic dysfunction, there was a difference in RV size and function between the two groups. Compared to the PH_PVD_ group, the PH_LHD_ group showed greater RV dilation (RVEDd 4.7 ± 0.7 vs. 4.03 ± 0.53; *p* = 0.0341) and RA dilation (RAVi 50.91 ± 21.56 vs. 30.6 ± 10.74; *p* = 0.0147). This was observed in the setting of similar markers of high RV afterload, as evidenced by comparable PASP (62.77 ± 17.18 vs. 57.41 ± 16.52; *p* = 0.354), IVS systolic septal flattening (100% vs. 69.23%; *p* > 0.9999), and the presence of RVOT PW doppler notching in both groups (100% vs. 69.23%; *p* = 0.1045). Additionally, the PH_LHD_ group demonstrated reduced markers of RV systolic function based on decreased TAPSE (1.33 ± 0.51 vs. 1.98 ± 0.62; *p* = 0.031) and RV S’ (8.97 ± 2.3 vs. 12.28 ± 2.90; *p* = 0.0054) when compared to the PH_PVD_ group.

### 3.2. Baseline Hemodynamics

At baseline, central hemodynamic parameters, including right atrial, pulmonary artery systolic, pulmonary artery diastolic, mean pulmonary artery, and pulmonary capillary wedge pressure, were higher in PH_LHD_ as compared to PH_PVD_ (Table 2). In contrast, flow and resistance data, including cardiac output, cardiac index, pulmonary vascular resistance, and systemic vascular resistance, were similar between both groups. Baseline HR and MAP were also similar. The PH_PVD_ group had a higher resting PAPi (4.65 ± 1.94 vs. 2.78 ± 0.79; *p* = 0.0093) and a lower RAP:PCWP ratio (0.54 ± 0.27 vs. 0.69 ± 0.18; *p* = 0.036) suggestive of preserved baseline RV systolic function.

### 3.3. Hemodynamic Response with Nitroprusside

**Figure 2:**
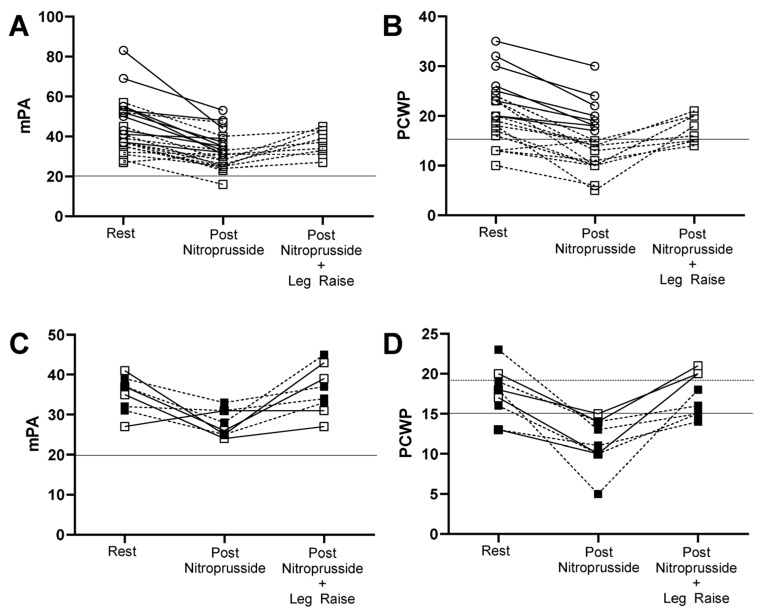
Course of mPA and PCWP response from rest, during nitroprusside infusion, and nitroprusside with leg raise where applicable. (**A**,**B**): mPA and PCWP response including leg raise, respectively, of (○ with solid line) PHLHD and (□ with dotted line) PHPVD. (**C**,**D**): mPA and PCWP response of PHPVD group, respectively, of (■ with dotted line) PHPVD group with PCWP ≤ 18 with nitroprusside and post leg raise and (□ with solid line) PHPVD > 18 with nitroprusside and post leg raise. Each line connects the same patient with each condition.

The dose of nitroprusside required to achieve the target blood pressure or PCWP was higher in the PH_LHD_ cohort (Table 1; 1.22 ± 0.40 vs. 0.84 ± 0.42; *p* = 0.0126). With the addition of nitroprusside, the intracardiac and intrapulmonary pressures remained higher in the PH_LHD_ cohort despite similar resting and post-nitroprusside systemic vascular resistance (SVR; Figure 2, Table 1 and Table S1). In contrast, the change in the filling pressures (Table 3) from pre to post nitroprusside decreased to a similar degree, except for ΔRAP. ΔRAP evidenced a greater change in the PH_LHD_ group (−5.80 ± 2.86 vs. −2.77 ± 1.36; *p* = 0.0092). Although the ΔPCWP decreased to a similar degree in both groups (−6.29 ± 4.25 mmHg vs. −5.10 ± 2.42; *p* = 0.4254), the mean PCWP in PH_PVD_ group decreased from 17.92 to below 15 with a mean decrease to 11.64 (*p* = 0.0004) and in the PH_LHD_ group from 25.6 to 20.5 (*p* = 0.0002). The ΔTPG pre and post nitroprusside showed minimal change in the PH_PVD_ group (−3.00 ± 5.67) as compared to the PH_LHD_ group (−10.00 ± 9.68; *p* = 0.0433), suggestive of a component of fixed pulmonary vascular disease in the PH_PVD_ group. The ΔPVR was concordant with the ΔTPG finding with a greater change in the ΔPVR PH_LHD_ (−3.57 ± 3.52) than the ΔPVR PH_PVD_ group (−1.08 ± 1.61; *p* = 0.0417). As compared to the PH_PVD_ group, the PH_LHD_ group showed an increase in cardiac output (CO), cardiac index (CI), stroke volume (SV), and stroke volume index (SVi) without a significant change in heart rate (HR). The baseline left ventricular transmural filling pressure (LVTMP) was similar in both groups (PH_PVD_ 8.57 ± 5.40 vs. PH_LHD_ 5.00 ± 5.10; *p* = 0.65). Post nitroprusside, the LVTMFP decreased in the PH_PVD_ group (8.57 ± 5.40 to 5.00 ± 2.42; *p* = 0.0123) and remained stable in the PH_LHD_ group (8.00 ± 5.10 to 8.70 ± 3.89; *p* = 0.6445) resulting in a greater change in ΔLVTMFP in the PH_PVD_ group (PH_PVD_ −3.57 ± 4.67 vs. PH_LHD_ 0.70 ± 3.74; *p* = 0.045).

### 3.4. Hemodynamic Response to Nitroprusside and Leg Raise

In the PH_PVD_ group (Figure 1, Table S1), nine patients met criteria with PCWP ≤ 15 mmHg and PVR ≥ 3 WU post nitroprusside for leg raise. Five patients in the PH_PVD_ group had PVR < 3 WU post nitroprusside, and, therefore, did not undergo leg raise. In the PHLHD group, two patients underwent leg raise during nitroprusside infusion as well. Within two minutes of leg raise, six patients maintained a PCWP ≤ 18 mmHg in the PH_PVD_ group (Figure 1 and Figure 2, Tables S2–S4). Consequently, these patients underwent treatment with pulmonary vascular-targeted therapy (Table 4 for Δdata, Table S5 for pre and post data). One of the six patients did not tolerate any PAH therapy due to medication side effects. The other five were started on PAH therapy and reassessed within 3 months with improvement in functional parameters as assessed by a 6MWD increase by >85 m with a concordant decrease by 1 WHO Functional Class in each patient. Right ventricular parameters improved as reflected in a decrease in %∆NT-proBNP from baseline. Each patient had an improvement in their REVEAL 2.0 Lite Risk Score by ≥4 with decrease from High risk to Intermediate or Low risk in four out of the five patients [23]. 

## 4. Discussion

In this small study, we demonstrate how nitroprusside plus leg raise can be utilized to differentiate precapillary from predominant postcapillary pulmonary hypertension in a cohort of patients that exhibit systemic hypertension at the time of right heart catheterization. Post nitroprusside infusion, the predominant precapillary PH group was defined by a decrease in PCWP ≤ 15 mmHg with persistent elevation of PVR ≥ 3 WU, i.e., the PH_PVD_ group. Pulmonary vascular-targeted therapy was well tolerated in the cohort of patients who had a PCWP ≤ 15 mmHg with infusion of nitroprusside and PCWP ≤ 18 mmHg with simultaneous nitroprusside infusion plus leg raise accompanied by improved functional parameters.

Although the total number of patients is low, the PCWP response between the three groups from post nitroprusside PCWP to post nitroprusside plus leg raise PCWP, i.e., PH_PVD_ with PCWP ≤ 18 mmHg with nitroprusside plus leg raise vs. PH_PVD_ with PCWP ≥ 18 mmHg with nitroprusside plus leg raise (PCWP 14.67 ± 2.66 vs. 20.33 ± 0.58; *p* = 0.119), and PH_LHD_ nitroprusside plus leg raise (24.5 ± 0.71; *p* = 0.009 [compared to both PH_PVD_ groups]), suggests that preload augmentation during nitroprusside infusion via passive leg raise can potentially help differentiate and identify patients that may tolerate PAH therapy. The PH_PVD_ patients that maintained a PCWP ≤ 18 mmHg with leg raise were patients with ILD, toxin-induced, or idiopathic PAH. The reversible rise in PCWP from increased LV afterload from systemic hypertension in this subgroup of the PH_PVD_ group may be stress-induced, i.e., anxiety, potentially holding AM medications including anti-hypertensives, and/or an early or less-advanced form of HFpEF. This shows the value of dynamic testing to phenotype patients. 

Nitroprusside is an arterial vasodilator and venodilator [24,25], both of which play an important role in reducing filling pressures [24]. In the left-sided circulation of the PH_PVD_ and PH_LHD_ groups with nitroprusside infusion, we noted a decrease in PCWP by a similar amount; however, the overall left-sided filling pressures were higher in the PH_LHD_ group despite similar baseline MAP and SVR that required higher doses of nitroprusside to achieve the same MAP and SVR post infusion. This is likely due to increased LV/LA noncompliance in the setting of higher vascular stiffness in a more severe HFpEF-dominant cpcPH phenotype [2,19,26]. In the right-sided circulation, the echocardiogram and RHC show a more advanced RV-dominant phenotype in the PH_LHD_ group at baseline with increased RV/RA dilation, increased RAP and PA pressures, and enhanced pericardial constraint compared to the PH_PVD_ group [27,28]. The difference in RV metrics is likely driven by long-standing elevated left-sided filling pressures resulting in an increased RV pulsatile load causing a disproportionate decrease in the pulmonary vascular compliance in a more advanced HFpEF phenotype [2,29]. There was an ~11% increase in CO, SV, and SVi in the PH_LHD_ group which is likely due to improved ventricular–arterial coupling from the decrease in the overall “vascular tone” on the basis that the heart rate remained stable. This improvement is similar to that seen in Schwartzenberg et al. of ~17% improvement with nitroprusside [19]. The minimal hemodynamic change in CO and SV suggests that the PH_LHD_ group are more likely to be operating closer to the flatter portion of their Starling curves and is similar to that seen in other studies with a cpcPH–HFpEF population [19,26].

Nitroprusside response and leg raise response have been studied separately in cpcPH and PAH cohorts previously, but not sequentially, making direct comparison of our results difficult. With respect to nitroprusside response, we noted similar overall decreases in filling pressures post nitroprusside; however, our PH_PVD_ and PH_LHD_ cohort both had higher baseline resting PVR > 5 compared to prior studies [19]. The PH_PVD_ TPG and PVR response was similar to that of a PAH cohort in that the TPG and PVR were stable to minimally changed with increase in heart rate during nitroprusside infusion, but was different in that the PCWP did not change in a PAH cohort [18]. Passive leg raise alone has been studied in HFpEF, cpcPH, and PAH [20,21]. As in prior studies, passive leg raise resulted in an increase in intracardiac filling pressures, especially in the PCWP in the setting of nitroprusside infusion. This suggests that despite nitroprusside venodilation, the preload augmentation with passive leg raise can still be effective. Larger studies will be needed to determine optimal PCWP cutoff values for sequential testing with nitroprusside and passive leg raise to differentiate pre- vs. postcapillary disease. 

## 5. Limitations

This study is limited by its retrospective nature, cross-sectional design, small sample size, and catheterization laboratory referral population. The sample is also limited to hypertensive patients who received nitroprusside, suggesting possible bias, although in our laboratory it is routine to administer nitroprusside to all patients that are hypertensive with elevated left heart filling pressures regardless of ejection fraction. Another potential limitation is the lack of a uniform criteria for MAP or systolic blood pressure (SBP) target with nitroprusside infusion in cpcPH patients. We targeted MAP and SBP reduction to normotension using nitroprusside, which resulted in slightly higher MAPs than in other studies, with the goal to lower the PCWP ≤ 15 mmHg rather than normalize PVR [18,19]. As this is the first published report to our knowledge of utilizing sequential testing using nitroprusside and leg raise in a cpcPH patient cohort, there is no established PCWP cutoff value for defining HFpEF in this setting. The sample size in this study was too small to establish any standards in this regard. However, we felt it was reasonable to extend the published cutoff value ≤18 mmHg as this was irrespective of diuretic use [21]. 

## 6. Conclusions

In this small pilot study, we show that nitroprusside combined with passive leg raise can be used to differentiate precapillary from predominant postcapillary PH in a cohort of patients with cpcPH that are hypertensive at the time of RHC. Pulmonary vascular-targeted therapy was well tolerated in the cohort of patients who had a decrease in PCWP ≤ 15 mmHg with infusion of nitroprusside and PCWP ≤ 18 mmHg with simultaneous nitroprusside infusion and leg raise with improved functional parameters on pulmonary vascular therapy. The findings need to be explored further in larger studies to define the group of patients that may derive benefit from a similar strategy, as well as cutoff values for abnormal PCWP with combined nitroprusside and passive leg raise.

## Figures and Tables

**Table 1:** Baseline Patient Demographic Data and Functional Parameters of PH_PVD_ versus PH_LHD_ group.

Characteristics	PH_PVD_	PH_LHD_	*p* Value
Total Number of Patients	*n* = 14	*n* = 10	
Female	10 (73.00%)	5 (50.00%)	0.2038
Age (Years)	75 (56–89)	74 (51–88)	0.8739
Nitroprusside Dose (mcg/kg/min)	0.84 ± 0.42	1.22 ± 0.40	0.0126
Comorbidities			
HFpEF	9 (64.20%)	9 (90.00%)	0.3408
BMI (kg/m^2^)	29 (17.77–45.21)	33 (21.45–60.45)	0.4031
CAD	7 (50.00%)	4 (40.00%)	0.6968
HTN	11 (78.60%)	10 (100.00%)	0.2391
DM II	7 (50.00%)	4 (40.00%)	0.6968
Atrial fibrillation/Flutter	6 (42.90%)	7 (70.00%)	0.2397
Lung Disease (COPD/ILD)	8 (57.10%)	4 (40.00%)	0.6802
OSA	6 (42.90%)	5 (50.00%)	0.9999
CKD	7 (50.00%)	1(10.00%)	0.0791
Autoimmune diseases	3 (21.40%)	0 (0%)	0.2391
PH Etiology			
Non-PAH †	2		
PAH			
Idiopathic-PAH	2		
ILD-PAH	2		
CTD-PAH	2		
Toxin-PAH	1		
Medications			
ARNi/ACEi/ARB	9 (64.20%)	8 (80.00%)	0.6529
MRA	10 (71.40%)	9 (90.00%)	0.3577
SGLT2i	2 (14.20%)	7 (70.00%)	0.0104
PDE5 inhibitor only	2 (14.20%)	1 (10.00%)	>0.9999
Echocardiographic Parameters			
	Mean ± STDev	Mean ± STDev	*p* Value
LVEF	63.46 ± 8.02	59.4 ± 5.91	0.1902
LAVi	37.04 ± 11.12	48.88 ± 31.86	0.4833
Diastolic Dysfunction (Grade I–III)	9 (64.20%)	8 (80.00%)	0.66
IVS Systolic Septal flattening	9 (69.23%)	7 (70.00%)	>0.9999
RVEDd (cm)—basal width	4.03 ± 0.52	4.7 ± 0.70	0.0341
RVOT PW doppler notch	9 (69.23%)	10 (100%)	0.1045
RA size (RAVi)	30.60 ± 10.74	50.91 ± 21.56	0.0147
PASP	57.41 ± 16.52	62.77 ±17.18	0.354
TAPSE	1.98 ± 0.62	1.33 ± 0.51	0.031
RV S′	12.28 ± 2.89	8.97 ± 2.30	0.0054

Notes: For nominal variables *n* (%), for continuous variables mean (range) standard deviation as applicable. Abbreviation: PH_PVD_, pulmonary hypertension-pulmonary vascular disease; PH_LHD_, pulmonary hypertension-left heart disease; HFpEF, heart failure with preserved ejection fraction; BMI, body mass index; CAD, coronary artery disease; HTN, hypertension; DM II, diabetes mellitus type 2; OSA, obstructive sleep apnea; CKD, chronic kidney disease; PAH, pulmonary arterial hypertension; CTD, connective tissue disease; ILD, interstitial lung disease; ARNi, angiotensin receptor/neprilysin inhibitor; ACEi, angiotensin-converting enzyme inhibitor; ARB, angiotensin receptor blocker; MRA, mineralocorticoid receptor antagonists; SGLT2i, sodium–glucose cotransporter-2 inhibitor; PDE5, Phosphodiesterase 5 Inhibitors; LVEF, left ventricular ejection fraction; IVS, interventricular septum; RVEDd, right ventricular end-diastolic diameter; RVOT, right ventricular outflow tract; RA, right atrium; RAVi, right atrial volume index; PASP, pulmonary arterial systolic pressure; TAPSE, tricuspid annular plane systolic excursion; RV S′, Right ventricular peak tricuspid annular systolic tissue velocity. † Non-PAH PH: Defined as combined pre- and postcapillary pulmonary hypertension (cpcPH) with normalization of PCWP with nitroprusside but no clear PAH etiology; this phenotype is distinguished from pulmonary arterial hypertension (PAH) by the presence of a significant postcapillary component, which was characterized during this study (see Figure 1).

**Table 2:** Baseline RHC Hemodynamic Parameters.

Resting	PH_PVD_	PH_LHD_	*p*-Value ^a^
	Mean ± STDev	Mean ± STDev	
RAP (mmHg)	9.35 † ± 3.56	17.6 ‡ ± 5.02	0.0002
PASP (mmHg)	64.00 † ± 16.71	84.9 ‡ ± 16.00	0.0044
PADP (mmHg)	25.00 † ± 5.87	38.3 ‡ ± 13.90	0.0006
mPAP (mmHg)	38.00 † ± 8.50	53.83 ‡± 13.70	0.0004
PCWP (mmHg)	17.92 † ± 4.57	25.6 ‡ ± 5.27	0.0009
DPG (mmHg)	7.07 ± 4.50	12.7 ± 13.22	0.5345
TPG (mmHg)	20.07 ± 7.96	28.23 ‡ ± 12.32	0.0865
PVR (WU)	5.33 † ± 2.62	7.71 ‡ ± 5.03	0.3784
SVR (mmHg⋅min⋅mL^−1^)	1990.61 † ± 332.13	1633.62 ‡ ± 647.98	0.4279
CO (L/min)	4.02 ± 1.04	4.24 ‡ ± 1.35	0.5938
CI (L/min/m^2^)	2.08 ± 0.56	2.11 ‡ ± 0.56	0.8894
SV (mL)	59.39 ± 12.29	59.66 ‡ ± 21.65	0.7638
SVi (mL/m^2^)	30.93 ± 7.55	30.18 ‡ ± 11.44	0.5458
HR (bpm)	67.71 † ± 9.11	72.6 ± 12.05	0.5159
MAP (mmHg)	106.35 † ± 12.72	104.10 ‡ ± 12.30	0.6555
PAPi	4.65 ± 1.94	2.78 ± 0.79	0.0093
RAP:PCWP	0.54 ± 0.27	0.69 ‡ ± 0.18	0.0356
LVTMFP	8.57 † ± 5.40	8.00 ± 5.10	0.6548

Notes: For nominal variables n (%), for continuous variables mean (range) standard deviation. Abbreviations: RAP, right atrial pressure; PASP, pulmonary artery systolic pressure; PADP, pulmonary artery diastolic pressure; mPAP, mean pulmonary artery pressure; PCWP, pulmonary capillary wedge pressure; DPG, diastolic pulmonary gradient; TPG, transpulmonary gradient; PVR, pulmonary vascular resistance; WU, wood units; SVR, systemic vascular resistance; CO, cardiac output; CI, cardiac index; SV, stroke volume; SVi, stroke volume index; HR, heart rate; MAP, mean arterial pressure; PAPi, pulmonary artery pulsatility index; LVTMFP, left ventricular transmural filling pressure. ^a^ *p*-value is comparison of resting hemodynamic parameters. † Denotes *p* < 0.05 between resting and post nitroprusside of PH_PVD_ of same variable, i.e., RAP, PASP, etc. ‡ Denotes *p* < 0.05 between resting and post nitroprusside of PH_LHD_ of same variable, i.e., RAP, PASP, etc.

**Table 3:** Changes in Hemodynamic Parameters in Response to Nitroprusside.

Parameter Δ	PH_PVD_	PH_LHD_	*p*-Value ^a^
	Mean ± STDev	Mean ± STDev	
ΔRAP (mmHg)	−2.77 ± 1.36	−5.80 ± 2.86	0.0092
ΔPASP (mmHg)	−16.14 ± 10.68	−21.90 ± 11.29	0.1847
ΔPADP (mmHg)	−5.86 ± 4.45	−11.70 ± 12.82	0.2387
ΔmPAP (mmHg)	−9.29 ± 5.96	−15.10 ± 11.19	0.2407
ΔPCWP (mmHg)	−6.29 ± 4.25	−5.10 ± 2.42	0.4254
ΔDPG (mmHg)	0.43 ± 4.78	−6.60 ± 11.47	0.0614
ΔTPG (mmHg)	−3.00 ± 5.67	−10.00 ± 9.68	0.0433
ΔPVR (WU)	−1.08 ± 1.61	−3.57 ± 3.52	0.0417
ΔSVR (mmHg⋅min⋅mL^−1^)	−578.43 ± 275.17	−629.59 ± 333.42	0.8315
ΔCO (L/min)	0.22 ± 0.71	0.55 ± 0.35	0.0468
ΔCI (L/min/m^2^)	0.17 ± 0.32	0.28 ± 0.16	0.0928
ΔSV (mL)	−1.83 ± 8.79	6.77 ± 5.88	0.0178
ΔSVi (mL/m^2^)	−0.33 ± 3.54	3.57 ± 2.95	0.0084
ΔHR (bpm)	5.07 ± 4.67	0.90 ± 5.24	0.0742
ΔMAP (mmHg)	−30.07 ± 12.07	−28.1 ± 13.26	0.8068
ΔPAPi	0.18 ± 1.24	1.04 ± 1.83	0.7961
ΔRAP:PCWP	0.02 ± 0.20	−0.12 ± 0.15	0.7961
ΔLVTMFP	−3.57 ± 4.67	0.70 ± 3.74	0.0454

Notes: For nominal variables n (%), for continuous variables mean (range) standard deviation. Abbreviations: Δ, Delta; RAP, right atrial pressure; PASP, pulmonary artery systolic pressure; PADP, pulmonary artery diastolic pressure; mPAP, mean pulmonary artery pressure; PCWP, pulmonary capillary wedge pressure; DPG, diastolic pulmonary gradient; TPG, transpulmonary gradient; PVR, pulmonary vascular resistance; WU, wood units; SVR, systemic vascular resistance; CO, cardiac output; CI, cardiac index; SV, stroke volume; SVi, stroke volume index; HR, heart rate; MAP, mean arterial pressure; PAPi, pulmonary artery pulsatility index; LVTMFP, left ventricular transmural filling pressure. ^a^*p*-value is comparison of change in hemodynamic parameters from rest to post nitroprusside of PH_LHD_ vs. PH_PVD_.

**Table 4:** PH_PVD_ response to PAH therapy within three months of initiation.

PH_PVD_ Patient	Etiology	Medication	Δ6MWD (m)	ΔWHO FC	ΔNT-proBNP (% Change from Baseline)	ΔREVEAL Lite 2.0 Risk Score ^a^	REVEAL Lite 2.0 Risk Status ^b^
Patient 1	ILD	Sildenafil 40 mg PO q8hrs +INH Treprostinil 64 mcg INH QID	+209	−1	−72%	−5	Intermediate
Patient 2	ILD	Sildenafil 60 mg PO q8hrs ^c^	+145	−1	−68%	−5	Intermediate
Patient 3	Toxin	Sildenafil 20 mg PO q8hrs ^c^	+130	−1	−45%	−4	High
Patient 4	Idiopathic	Sildenafil 60 mg q8hr	+244	−1	−36%	−5	Low
Patient 5	Idiopathic	Sildenafil 20 mg PO q8hrs	+85	−1	−84%	−5	Low

Abbreviations: INH, inhaled; Δ, Delta; PVD, Pulmonary vascular disease; PH, Pulmonary hypertension; ILD, interstitial lung disease; PO, Per os (by mouth); q8hr, every eight hours; mcg, micrograms; 6MWD, six minute walk distance; WHO FC, World Health Organization Functional Class; m, meters; NT-proBNP, N-terminal-pro Brain Natriuretic Peptide; REVEAL Lite 2.0 Risk Score, Registry to Evaluate Early and Long Term PAH Disease Management Lite 2.0 Risk Score [23]. ^a^ All five patients had an initial REVEAL Lite 2.0 Score ≥ 9. The reported value is the decrease from baseline, and NOT the final score. ^b^ All five patients were High Risk prior to initiation of PAH therapy. The reported status is after initiation of PAH therapy. ^c^ Patient refused additional PAH therapy.

## Data Availability

Data supporting this study are available upon request. Please contact the corresponding author.

## Supplementary Materials

The following supporting information can be downloaded at: https://www.mdpi.com/article/10.3390/jcdd11040124/s1. Table S1: RHC Hemodynamic Parameters in Response to Nitroprusside. Table S2: RHC Hemodynamic Parameters in Response to Nitroprusside Administration and Leg Raise Maneuver: PH_PVD_ with Leg Raise PCWP ≤ 18 mmHg vs. PH_PVD_ with Leg Raise PCWP > 18 mmHg vs. PH_LHD_. Table S3: Hemodynamic Changes in RHC Parameters in Response to Nitroprusside Administration and Leg Raise Maneuver: PH_PVD_ with Leg Raise PCWP ≤ 18 mmHg vs. PH_PVD_ with Leg Raise PCWP > 18 mmHg vs. PH_LHD_. Table S4: Comparison of Echocardiographic and Functional Parameters in the Leg Raise Cohort: PH_PVD_ with Leg Raise PCWP ≤ 18 mmHg vs. PH_PVD_ with Leg Raise PCWP > 18 mmHg vs. PH_LHD_. Table S5: PH_PVD_ response to PAH therapy within three months of initiation (Raw Data).
