# Supplementary material for: Nitroprusside Combined with Leg Raise at the Time of Right Heart Catheterization to Differentiate Precapillary from Other Hemodynamic Forms of Pulmonary Hypertension: A Single-Center Pilot Study
Source: J Cardiovasc Dev Dis. 2024 Apr 19;11(4):124. doi: 10.3390/jcdd11040124 (PMC11050046; doi:10.3390/jcdd11040124)
Supplement: Supplementary file 1 [file jcdd-11-00124-s001.zip › jcdd-2861951-supplementary.pdf]

Supplemental Tables:

Supplementary Table S1: RHC Hemodynamic Parameters in Response to Nitroprusside

Parameter	PH_{PVD} Mean ± STDev	PH_{LHD} Mean ± STDev	P-value[a]
RAP (mmHg)	6.92 ± 2.81	11.80 ± 4.57	0.0132
PASP (mmHg)	47.85 ± 15.82	63.00 ± 10.83	0.0045
PADP (mmHg)	19.14± 4.99	26.60 ± 6.28	0.0024
mPAP (mmHg)	28.71 ± 7.62	38.73 ± 7.43	0.0012
PCWP (mmHg)	11.64 ± 3.34	20.50 ± 4.01	0.0001
DPG (mmHg)	7.50 ± 4.27	6.10 ± 5.57	0.3435
TPG (mmHg)	17.07 ± 7.08	18.23 ± 6.85	0.7632
PVR (WU)	4.24 ± 1.92	4.13 ± 1.91	0.8793
SVR (mmHg·min·mL-1)	1449.80 ± 362.16	1169.86 ± 413.40	0.2030
CO (L/min)	4.28 ± 1.47	4.79 ± 1.41	0.3349
CI (L/min/m2)	2.25 ± 0.75	2.39 ± 0.56	0.3669
SV (ml)	58.40 ± 17.10	66.43 ± 21.15	0.2839
SVi (ml/m^2)	30.85 ± 9.37	33.74 ± 11.32	0.6049
HR (bpm)	72.79 ± 8.78	73.50 ± 15.11	0.6139
MAP (mmHg)	76.29 ± 10.10	76.00 ± 11.26	0.8517
PAPi	4.58 ± 1.95	3.82 ± 2.34	0.3328
RAP: PCWP	0.58 ± 0.17	0.58 ± 0.20	0.9428
LVTMFP	5.15 ± 2.44	8.70 ± 3.89	0.0387

Notes: For nominal variables n (%), for continuous variables mean (range) standard deviation. Abbreviations: RAP, right atrial pressure; PASP, pulmonary artery systolic pressure; PADP, pulmonary artery diastolic pressure; mPAP, mean pulmonary artery pressure; PCWP, pulmonary capillary wedge pressure; DPG, diastolic pulmonary gradient; TPG, transpulmonary gradient; PVR, pulmonary vascular resistance; WU, wood units; SVR, systemic vascular resistance; CO, cardiac output; CI, cardiac index; SV, stroke volume; HR, heart rate; MAP, mean arterial pressure; PAPi, pulmonary artery Pulsatility index; LVTMFP, left ventricular transmural filling pressure

[a] p-value is comparison of post nitroprusside PH_{LHD} vs. post nitroprusside PH_{PVD} hemodynamic parameters

Supplementary Table S2: RHC Hemodynamic Parameters in Response to Nitroprusside Administration and Leg Raise Maneuver: PH_{PVD} with Leg Raise PCWP ≤ 18mmHg vs. PH_{PVD} with Leg Raise PCWP > 18mmHg vs. PH_{LHD}

	PH_{PVD} Group with PCWP ≤ 18mmHg Post Leg Raise Maneuver N=6			PH_{PVD} Group with PCWP > 18mmHg Post Leg Raise Maneuver N=3			PH_{LHD} Group with PCWP > 18 mmHg Post Leg Raise Maneuver N=2		
	Rest	Post Nipride	Post Leg Raise	Rest	Post Nipride	Post Leg Raise	Rest	Post Nipride	Post Leg Raise
	Mean ± STDev	Mean ± STDev	Mean ± STDev	Mean ± STDev	Mean ± STDev	Mean ± STDev	Mean ± STDev	Mean ± STDev	Mean ± STDev
**RAP (mmHg)**	8.67 ± 3.14	6.33 ± 2.42	10.5 ± 4.32	9.33 ± 3.06	6 ± 1.73	12.33 ± 2.08	21 ± 1.41	15 ± 1.41	23 ± 5.66
**PASP (mmHg)**	66.33 ± 17.91	51.83 ± 11.43	61.67 ± 12.36	60.67 ± 13.32	44.00 ± 6.56	61.00 ± 11.53	63 ± 7.07	52 ± 5.66	60 ± 14.14
**PADP (mmHg)**	24.5 ± 6.69	19.33 ± 4.41	24.17 ± 5.38	22.33 ± 3.79	18.67 ± 3.21	25.67 ± 4.04	28 ± 2.83	24 ± 0	30 ± 2.83
**mPAP (mmHg)**	38.44 ± 9.74	30.17 ± 5.94	36.67 ± 6.55	35.11 ± 6.94	27.11 ± 3.10	37.44 ± 6.26	39.67 ± 4.24	33.33 ± 1.89	40 ± 7.07
**PCWP (mmHg)**	17 ± 3.85	10.50 ± 3.15	14.67 ± 2.66†	18.33 ± 1.53	13 ± 2.65	20.33 ± 0.58†	22.5 ± 3.54‡	19 ± 1.41	24.5 ± 0.71†‡
**DPG (mmHg)**	7.5 ± 3.62	8.83 ± 4.12	9.50 ± 3.08	4.00 ± 2.65	5.67 ± 5.03	5.33 ± 3.79	5.5 ± 0.71	5 ± 1.41	5.5 ± 2.12
**TPG (mmHg)**	21.44 ± 6.72	19.67 ± 4.88	22.00 ± 4.51	16.78 ± 5.62	14.11 ± 5.68	17.11 ± 5.82	17.17 ± 0.71	14.33 ± 0.47	15.5 ± 6.36

Abbreviations: RAP, right atrial pressure; PASP, pulmonary artery systolic pressure; PADP, pulmonary artery diastolic pressure; mPAP, mean pulmonary artery pressure; PCWP, pulmonary capillary wedge pressure; DPG, diastolic pulmonary gradient; TPG, transpulmonary gradient;

† Denotes P<0.05 between each group with same condition, ie post leg raise PCWP in each subgroup

† Denotes P<0.05 between resting PH_{PVD} PCWP <=18 post leg raise vs PH_{PVD} PCWP >18 post LR group of same condition, ie PCWP at rest in each group

‡ Denotes P<0.05 between PH_{PVD} groups vs PH_{LHD} group with PCWP>18 group in same condition, ie PCWP at rest conditions

Supplementary Table S3: Hemodynamic Changes in RHC Parameters in Response to Nitroprusside Administration and Leg Raise Maneuver: PH_{PVD} with Leg Raise PCWP ≤ 18mmHg vs. PH_{PVD} with Leg Raise PCWP > 18mmHg vs. PH_{LHD}

**Δ Post Nitroprusside - Rest**			
	**PH_{PVD} with PCWP ≤ 18mmHg Post Leg Raise**<br>**Mean ± STDev**	**PH_{PVD} with PCWP > 18mmHg Post Leg Raise**<br>**Mean ± STDev**	**PH_{LHD} with PCWP > 18mmHg Post Leg Raise**<br>**Mean ± STDev**
**ΔRAP (mmHg)**	-2.33 ± 0.82	-3.33 ± 2.08	-6.00 ± 0
**ΔPASP (mmHg)**	-14.5 ± 11.69	-16.67 ± 17.90	-11.00 ± 1.41
**ΔPADP (mmHg)**	-5.17 ± 2.99	-3.67 ± 6.51	-4.00 ± 2.83
**ΔmPAP (mmHg)**	-8.28 ± 5.52	-8.00 ± 10.02	-6.33 ± 2.36
**ΔPCWP (mmHg)**	-6.50 ± 4.23	-5.33 ± 2.08	-3.5 ± 2.12
**ΔDPG (mmHg)**	1.33 ± 4.13	1.67 ± 7.37	-0.50 ± 0.71
**ΔTPG (mmHg)**	-1.78 ± 4.64	-2.67 ± 11.27	-2.83 ± 0.24
**ΔPVR (WU)**	-1.02 ± 2.03	-0.69 ± 2.21	
**ΔSVR (mmHg·min·mL-1)**	-557.47 ± 352.52	-543.34 ± 185.59	
**Δ CO (L/min)**	0.16 ± 0.48	-0.30 ± 0.60	
**Δ CI (L/min/m2)**	0.10 ± 0.28	0.00 ± 0.10	
**ΔSV (ml)**	-2.80 ± 6.43	-7.45 ± 13.41	
**ΔSVi (ml/m^2)**	-1.58 ± 3.77	-1.25 ± 3.50	
**ΔHR (bpm)**	5.50 ± 5.65	2.67 ± 5.86	
**ΔMAP (mmHg)**	-23.83 ± 9.13	-37.33 ± 14.01	
**ΔPAPi**	0.08 ± 1.29	0.41 ± 1.40	
**ΔRAP: PCWP**	0.09 ± 0.23	-0.04 ± 0.16	
**ΔLVTMFP**	-4.17 ± 4.71	-2.00 ± 3.61	

**Δ Leg Raise - Resting**			
	**PH_{PVD} with PCWP ≤ 18mmHg Post Leg Raise**<br>**Mean ± STDev**	**PH_{PVD} with PCWP > 18mmHg Post Leg Raise**<br>**Mean ± STDev**	**PH_{LHD} with PCWP > 18mmHg Post Leg Raise**<br>**Mean ± STDev**
**Δ RAP (mmHg)**	1.83 ± 3.87	3.00 ± 1.73	2.00 ± 4.25
**Δ PASP (mmHg)**	-4.67 ± 11.57	0.33 ± 4.04	-3.00 ± 7.01
**Δ PADP (mmHg)**	-0.33 ± 5.85	3.33 ± 0.58	2.00 ± 0.00
**Δ mPAP (mmHg)**	-1.78 ± 7.71	2.33 ± 1.00	0.33 ± 2.83
**Δ PCWP (mmHg)**	-2.33 ± 3.20	2.00 ± 1.00	2.00 ± 2.83
**Δ DPG (mmHg)**	2.00 ± 3.22	1.33 ± 1.15	0.00 ± 2.83
**Δ TPG (mmHg)**	0.56 ± 4.98	0.33 ± 1.00	-1.67 ± 5.67
**ΔPVR (WU)**	NR	NR	NR

Δ Leg Raise - Nitroprusside

	PH_{PVD} with PCWP ≤ 18mmHg Post Leg Raise<br>Mean ± STDev	PH_{PVD} with PCWP > 18mmHg Post Leg Raise<br>Mean ± STDev	PH_{LHD} with PCWP > 18mmHg Post Leg Raise<br>Mean ± STDev
**Δ RAP (mmHg)**	4.17 ± 3.54	6.33 ± 2.31	8.00 ± 4.24
**Δ PASP (mmHg)**	9.83 ± 10.70	17.00 ± 14.93	8.00 ± 8.49
**Δ PADP (mmHg)**	4.83 ± 3.49	7.00 ± 6.56	6.00 ± 2.83
**Δ mPAP (mmHg)**	6.50 ± 5.35	10.33 ± 9.29	6.66 ± 5.19
**Δ PCWP (mmHg)**	4.17 ± 4.62	7.33 ± 2.52	5.50 ± 0.71
**Δ DPG (mmHg)**	0.67 ± 2.25	-0.33 ± 8.5	0.5 ± 3.54
**Δ TPG (mmHg)**	2.33 ± 1.33	3.00 ± 11.36	1.17 ± 5.89
**ΔPVR (WU)**	NR	NR	NR

Δ: delta; RAP, right atrial pressure; PASP, pulmonary arterial systolic pressure; PADP, pulmonary artery diastolic pressure; mPAP, mean pulmonary arterial pressure; PCWP, pulmonary capillary wedge pressure: DPG, diastolic pulmonary gradient; TPG, trans pulmonary gradient; PVR, pulmonary vascular resistance; SVR, systemic vascular resistance; CO, cardiac output; CI, cardiac index; SV, stroke volume; SVi, stroke volume index; HR, heart rate; MAP, mean arterial pressure; PAPi, pulmonary arterial pulsatility index; LVTMFP, left ventricular transmural filling pressure.

Supplementary Table S4: Comparison of Echocardiographic and Functional Parameters in the Leg Raise Cohort: PH_{PVD} with Leg Raise PCWP ≤ 18mmHg vs. PH_{PVD} with Leg Raise PCWP > 18mmHg vs. PH_{LHD}

Parameters	PH_{PVD} with Leg Raise PCWP ≤ 18mmHg<br><br>Mean ± STDev	PH_{PVD} with Leg Raise PCWP > 18mmHg<br><br>Mean ± STDev	PH_{LHD}<br><br>Mean ± STDev
Echocardiographic			
LVEF	64.5 ± 9.12	63.33 ± 7.02	65 ± 1.41
LVOT VTI	21.55 ± 6.31	21.27 ± 5.54	15.1 ± 5.37
LAVi	34.72 ± 10.04	38.17 ± 16.85	70.9 ± 72.27
E/A	1.65 ± 0.91	NR	NR
E' (m/s)	7.30 ± 2.47	6.5 ± 0.56	8.9 ± 0.85
E/e'	15.97 ± 7.18	12.7 ± 2.72	10.9 ± 1.41
Diastolic dysfunction	3 (50%)	1 (33.33%)	1 (50%)
Septal flattening	5 (83.33%)	2 (66.67%)	1 (50%)
RVEDd (cm) - basal length	4.22 ± 0.66	3.94 ± 0.29	5.55 ± 0.78
RVOT notch	4 (66.67%)	2 (66.67%)	2 (66.67%)
RVOT VTI (cm)	16.13 ± 4.55	15.68 ± 1.19	11.25 ± 5.73
RA size (RAVi)	35.73 ± 12.43	24.9 ± 10.09	57.3 ± 15.7
PASP	59.8 ± 17.51	47.33 ± 21.73	51 ± 21.21
TAPSE	2.03 ± 0.61	2.43 ± 0.71	1.25 ± 0.35
RV S'	13.85 ± 3.59	11.23 ± 1.58	6.8 ± 0.57
Pulmonary Function Tests			
FEV1 (%)	68.67 ± 24.59	90 ± 25.46	91 ± 31.11
FVC (%)	73.67 ± 17.84	93 ± 7.07	84.5 ± 26.16
FEV1/FVC (%)	68.5 ± 16.53	68 ± 14.14	76.5 ± 3.54
TLC (%)	79.2 ± 11.12	NR	88.5 ± 27.58
RV (%)	81.2 ± 36.79	NR	94 ± 41.01
RV/TLC (%)	1.01 ± 0.37	NR	1.04 ± 0.14
DLCO adjusted	47.6 ± 23.67	69.5 ± 10.61	75.5 ± 10.61

Abbreviations: LVEF, left ventricular ejection fraction; LVOT, left ventricular outflow tract; LAVi: left atrial volume index; E, early diastolic transmitral flow velocity; A, late diastolic transmitral flow velocity; E′ early diastolic tissue velocity, e′, early diastolic mitral annular velocity; IVS, interventricular septum; RVEDd, right ventricular end-diastolic diameter;

RVOT, right ventricular outflow tract; VTI, velocity time integral; RA, right atrium; RAVi, right atrial volume index; PASP, pulmonary arterial systolic pressure; TAPSE, tricuspid annular plane systolic excursion; RV, right ventricle; S′, peak tricuspid annular systolic tissue velocity; FEV1, forced expiratory volume in the first second; FVC, forced vital capacity; TLC, total lung capacity; RV*, residual volume; DLCO, diffusing capacity of the lungs for carbon monoxide.

Supplementary Table S5: PH_{PVD} response to PAH therapy within three months of initiation (Raw Data)

Patient	Etiology of PVD	PH Therapy added	Pre-PH therapy 6MWT (m)	Post-PH therapy 6MWT (m)	Pre-PH therapy NT-BNP (pg/mL)	Post-PH therapy NT-BNP (pg/mL)	Pre-PH therapy FC	Post-PH therapy FC	Pre-PH therapy REVEAL Lite 2.0 risk score	Post-PH therapy REVEAL Lite 2.0 risk score
**1**	ILD	Sildenafil 40 mg PO q8hrs +INH Treprostinil 64 mcg INH QID	121	330	15192	4236	III	II	12	7
**2**	ILD	Sildenafil 60 mg PO q8hrs[a]	40	185	3298	1046	III	II	11	6
**3**	Toxin	Sildenafil 20 mg PO q8hrs[a]	50	180	35000	19109	IV	III	12	8
**4**	Idiopathic.	Sildenafil 60 mg q8hr	61	305	235	151	III	II	9	4
**5**	Idiopathic.	Sildenafil 20 mg PO q8hrs	303	388	5019	799	III	II	10	5

Abbreviations: PVD: Pulmonary vascular disease; PH: Pulmonary hypertension; ILD, interstitial lung disease; INH: Inhaled; PO: Per os (by mouth); q8hr: every eight hours; mcg: micrograms; 6MWD, six minute walk distance; WHO FC, World Health Organization Functional Class; m: meters; NT-proBNP, N-terminal-pro Brain Natriuretic Peptide; REVEAL Lite 2.0 Risk Score, Registry to Evaluate Early and Long Term PAH Disease Management Lite 2.0 risk score [23].

[a]Patient refused additional therapy
